# Personal

**Published:** 1892-08

**Authors:** 


### Personal.

LEWIS S. McMURTRY, A. M., M. D., of Louisville, Ky., has been elected to the Chair of Gynecology in the Hospital College of Medicine, which is the Medical Department of the Central University of Kentucky.

To say that this is a most fitting appointment for the college to make, is only to repeat what everybody who knows Dr. McMurtry, will assent to. The advantages to the college will be very great in having so experienced a teacher, a man of so many qualities — social, scientific, and professional — in its faculty.

Dr. McMurtry will have agreeable associates in the college, which is one of the strongest in the south. The Hospital College of Medicine has taken a foremost stand with reference to advanced medical education, and its President, Dr. Dudley S. Reynolds, is, perhaps, the most conspicuous champion of the cause in the south. We shall take pleasure in soon recording Kentucky among the list of States with separate Medical Examining and Licensing Boards.

---

DR. ALBERT L. GIHON, of New York City, Medical Director U. S. Navy, delivered the address on General Medicine at the recent meeting of the American Medical Association, in Detroit. Dr. Gihon assumed the responsibilities of this duty on short notice, owing to an affliction which had befallen the regular appointee, and it is not an exaggeration to affirm that a better selection could not easily have been made. The subject of the address was Intellectual Progress in Medicine, which was handled with exceptional ability, and received the approbation of the entire assemblage, that culminated in a unanimous vote of thanks to the distinguished author.

---

Dr. Hunter McGuire, of Richmond, Va., was elected President of the American Medical Association, at Detroit, to serve for the ensuing year. This honor could not easily have fallen upon more competent shoulders or to the hands of a more popular man. Dr. McGuire has a strong following of friends throughout the south and southwest that are only warmer in their admiration than those of the north and northeast, because they are nearer to him geographically. The hearts of all, whether residing north, south, east, or west, are equally warm in their manifestations of approval of Dr. McGuire's election.

---

Dr. R. Stansbury Sutton, of Pittsburg, Pa., accompanied by his daughter, remained a night in Buffalo, in July, on his way to Penetanguishane, Ont., Canada, where he will spend a month's vacation. Dr. Sutton has lately been appointed gynecologist to the Allegany Hospital, which has been especially fitted up with a modern operating room for his convenience. This, together with his private hospital, gives Dr. Sutton ample facilities for the continuance of the practice of abdominal surgery, for which he has a record of success which is among the first in this country.

---

Dr. Edward R. Palmer, of Louisville, was elected President of the American Association of Andrology and Syphilology. This is a deserved recognition of the talent and skill of one of the most distinguished members of this association. We predict a successful year for the society under his administration.

---

Dr. Henry J. Mulford announces his removal from 415 Delaware avenue to the Fillmore, 107 Delaware avenue, Buffalo, where he will continue the special practice of laryngology.

---

Dr. A. Walter Suiter, of Herkimer, has been appointed consulting physician to the New Faxton Hospital, at Utica, N. Y.

